# Comparison of Deep Learning and Traditional Machine Learning Models for Predicting Mild Cognitive Impairment Using Plasma Proteomic Biomarkers

**DOI:** 10.3390/ijms26062428

**Published:** 2025-03-08

**Authors:** Kesheng Wang, Donald A. Adjeroh, Wei Fang, Suzy M. Walter, Danqing Xiao, Ubolrat Piamjariyakul, Chun Xu

**Affiliations:** 1 Department of Biobehavioral Health & Nursing Science, College of Nursing, University of South Carolina, Columbia, SC 29208, USA; 2 Department of Epidemiology and Biostatistics, Arnold School of Public Health, University of South Carolina, Columbia, SC 29208, USA; 3 Lane Department of Computer Science & Electrical Engineering, West Virginia University, Morgantown, WV 26506, USA; don@csee.wvu.edu; 4 West Virginia Clinical and Translational Science Institute, Morgantown, WV 26506, USA; fangweihb@gmail.com; 5 School of Nursing, Health Sciences Center, West Virginia University, Morgantown, WV 26506, USA; swalters@hsc.wvu.edu (S.M.W.); ubolrat.piamjariyakul@hsc.wvu.edu (U.P.); 6 Department of STEM, School of Arts and Sciences, Regis College, Weston, MA 02493, USA; danqing.xiao@regiscollege.edu; 7 Department of Health and Biomedical Sciences, College of Health Professions, University of Texas Rio Grande Valley, Brownsville, TX 78520, USA

**Keywords:** Alzheimer’s disease, mild cognitive impairment, plasma proteomics, machine learning, deep learning, deep neural network, bioinformatics, pathway

## Abstract

Mild cognitive impairment (MCI) is a clinical condition characterized by a decline in cognitive ability and progression of cognitive impairment. It is often considered a transitional stage between normal aging and Alzheimer’s disease (AD). This study aimed to compare deep learning (DL) and traditional machine learning (ML) methods in predicting MCI using plasma proteomic biomarkers. A total of 239 adults were selected from the Alzheimer’s Disease Neuroimaging Initiative (ADNI) cohort along with a pool of 146 plasma proteomic biomarkers. We evaluated seven traditional ML models (support vector machines (SVMs), logistic regression (LR), naïve Bayes (NB), random forest (RF), k-nearest neighbor (KNN), gradient boosting machine (GBM), and extreme gradient boosting (XGBoost)) and six variations of a deep neural network (DNN) model—the DL model in the H2O package. Least Absolute Shrinkage and Selection Operator (LASSO) selected 35 proteomic biomarkers from the pool. Based on grid search, the DNN model with an activation function of “Rectifier With Dropout” with 2 layers and 32 of 35 selected proteomic biomarkers revealed the best model with the highest accuracy of 0.995 and an F1 Score of 0.996, while among seven traditional ML methods, XGBoost was the best with an accuracy of 0.986 and an F1 Score of 0.985. Several biomarkers were correlated with the *APOE*-*ε4* genotype, polygenic hazard score (PHS), and three clinical cerebrospinal fluid biomarkers (Aβ42, tTau, and pTau). Bioinformatics analysis using Gene Ontology (GO) and Kyoto Encyclopedia of Genes and Genomes (KEGG) revealed several molecular functions and pathways associated with the selected biomarkers, including cytokine-cytokine receptor interaction, cholesterol metabolism, and regulation of lipid localization. The results showed that the DL model may represent a promising tool in the prediction of MCI. These plasma proteomic biomarkers may help with early diagnosis, prognostic risk stratification, and early treatment interventions for individuals at risk for MCI.

## 1. Introduction

Alzheimer’s disease (AD) is the most common cause of major neurocognitive disorder (dementia), which is characterized by progressive loss of memory and cognitive functions [1]. In 2024, AD and related forms of dementia affected 6.9 million Americans aged 65 and older, a number that is expected to increase to 13.8 million by 2060 [2]. AD is an age-related neurodegenerative disorder that is categorized by the progressive accumulation in the brain parenchyma of β-amyloid Aβ plaques (Aβ peptides) and neurofibrillary tangles (tau protein) [3,4,5]. The etiology of AD may start several years or decades before the onset of clinical symptoms; therefore, early diagnosis is a key element in managing AD progression [5,6,7,8].

AD is recognized as a disease that occurs along a symptomatic and chronological spectrum with phases of preclinical AD, amnestic mild cognitive impairment (MCI), and fully developed AD [9]. MCI is often considered a transitional stage between normal aging and AD. A previous study has shown that approximately 50% of patients with MCI develop AD within 5 years of diagnosis [10]. Regarding potential sources of MCI, recent studies have reported the association between several kinds of biomarkers and MCI, including apolipoprotein E (*APOE*) genotypes, Aβ plaques (Aβ42), pathologic tau (comprising total tau (tTau) and phosphorylated tau (pTau)), and neurodegenerative injury [5]. Changes in CSF reflect biochemical changes in the brain, and three commonly used key biomarkers in diagnosing AD are Aβ_42_, tTau, and pTau. One study found decreased CSF-Aβ over 25 years before the onset of AD [3]. Another study used CSF protein levels to show that AD pathophysiological processes may start before aggregated amyloid can be detected [11]. Thus, current mainstream detection tools mainly rely on analyzing CSF. However, brain-imaging tools using CSF are often extremely costly, and the diagnostic procedures involving CSF collection are invasive, adding to the discomfort and potential risks for patients. These factors contribute to the challenges surrounding the widespread adoption and accessibility of CSF-based diagnostic tools. For example, blood-based biomarkers could potentially aid early diagnosis as well as recruitment for clinical trials [5,12,13,14]. As shown in another example, proteomic analysis is capable of identifying both biological processes and altered signaling pathways during the pre-symptomatic phase of AD, even 20–30 years before the appearance of the first clinical symptoms of AD or other dementia-associated traits [14,15,16,17,18,19,20,21,22]. Therefore, blood proteomics may pave the way for the development of accurate, cost-effective, and minimally invasive AD diagnostics and screening tools for individuals at high risk. There is a critical need to identify noninvasive tools for early detection and disease progression.

In analyzing the association between the biomarkers and MCI and AD, machine learning (ML) methods show promise to translate univariate biomarker findings into clinically useful multivariate decision support systems. Several ML technologies have been used to enhance the diagnosis and prognosis of AD, such as logistic regression (LR), naive Bayes (NB), random forest (RF), decision trees (DT), k-nearest neighbor (KNN), gradient boosting machine (GBM), extreme gradient boosting (XGBoost), and support vector machines (SVM) [14,22,23,24,25,26,27,28,29,30]. In recent years, deep learning (DL) techniques have become increasingly popular in AD research, such as early diagnosis. For example, deep neural networks (DNNs), stacked autoencoder (SAE) neural networks, and convolutional neural networks (CNNs) [27,31,32,33] have been reported to be more accurate for AD diagnosis than conventional ML models [25,27,34,35,36,37]. However, DL-based studies in this field are still in their early stages, and further studies should incorporate different information sources [28].

Recently, while previous studies have shown that proteomic analyses with ML or DL models can reveal altered biological processes and heterogeneity in the presence of AD and other types of dementia [21,23,25,27,28,29,30,33,38,39,40,41,42], no study has focused on comparing ML and DL models when classifying the appearance of MCI using plasma proteomic biomarkers. Furthermore, proteomic biomarkers are abundant, and parts of these biomarkers are correlated with one another, which may be due to shared pathways or regulatory mechanisms. Developing ML/DL tools requires a critical consideration of the specific features used in the analysis. It is essentially the process of identifying the informative and relevant features from a larger collection of features, leading to an improved characterization of the underlying patterns and relations [43,44]. Feature selection algorithms are important in ML as they not only reduce the dimensionality of the feature space but also can reveal the most relevant features without losing too much information [45,46,47].

This study aimed to (1) compare seven traditional ML models (SVMs, LR, NB, RF, KNN, GBM, and XGBoost) and six variations of a DNN model—the DL model in the H2O package [48]; (2) predict MCI using plasma proteomic biomarkers; and (3) assess the functional relevance of the selected plasma proteomic biomarkers. Using the Alzheimer’s Disease Neuroimaging Initiative (ADNI) database (adni.loni.usc.edu), the findings revealed that application of a DL model may represent a promising tool in the prediction of MCI. These plasma proteomic biomarkers may help with early diagnosis, prognostic risk stratification, and early treatment interventions for individuals at risk for MCI.

## 2. Results

### 2.1. Descriptive Statistics

Table 1 displays characteristics of the 239 selected adults in this study. The results from either the *t*-test or Chi-square test revealed that age and education were not associated with MCI (*p* > 0.05); however, a significant association was observed for gender and *APOE-ε4* allele with MCI (*p* = 0.0334 and *p* < 0.0001, respectively) (Table 1). The MCI group had a lower mean value in the Aβ42 but higher PHS, tTau, and pTau scores (all *p* values < 0.0001).

### 2.2. Feature Selection and Resampling

**Figure 1:**
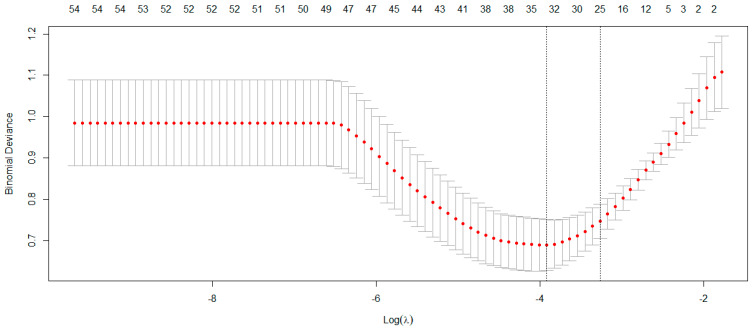
The LASSO method selected 35 biomarkers on the optimal parameter ln(λ) = −3.92.

Table 2 lists the feature selection package, algorithm, and extracted plasma proteomic biomarkers based on the Least Absolute Shrinkage and Selection Operator (LASSO) [49]. The LASSO effectively identified 35 plasma proteomic biomarkers based on optimal parameter ln(λ) = −3.92 (Figure 1). In addition, among 35 biomarkers selected by LASSO, 23 biomarkers were significantly associated with MCI using an independent *t*-test (Table S1). As there was an imbalance number between the two levels of MCI status, the “both” resampling method in random over-sampling examples (ROSE) [50], a bootstrap-based technique that aids the task of binary classification in the presence of rare classes, was used to generate the approximately balanced data, including 118 CN and 122 MCI individuals.

### 2.3. Machine Learning (ML) and Deep Learning (DL) Performance

**Figure 2:**
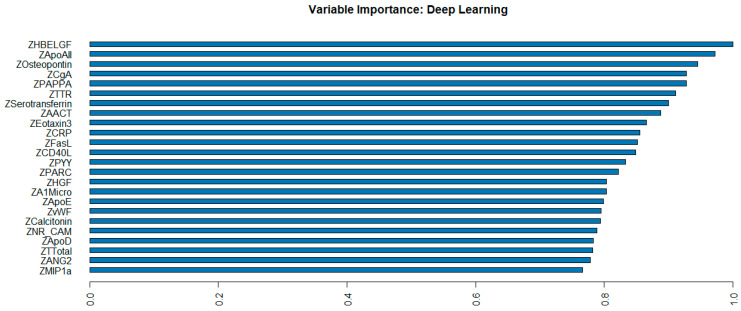
Variable importance of deep learning model with “Rectifier With Dropout” using 2 hidden layers of 32 proteomic biomarkers.

**Figure 3:**
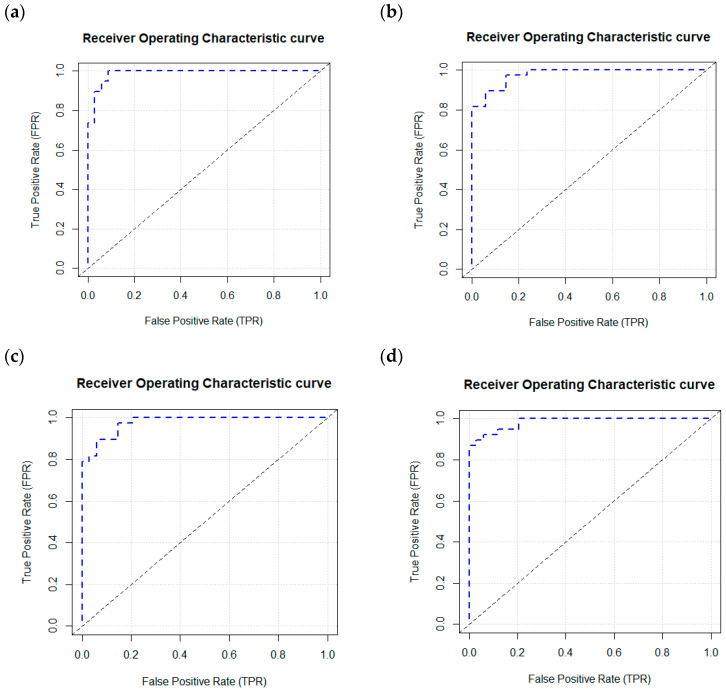
AUC curves in the validation data using a deep learning model with an H2O package. H2O. (**a**) Deep learning model using a Rectifier Activation Function with Dropout, 2 layers, and 32 inputs. (**b**) Deep learning model using a Tanh Activation Function with Dropout, 3 layers, and 32 inputs. (**c**) Deep learning model using a Tanh Activation Function with Dropout, 3 layers, and 33 inputs. (**d**) Deep learning model using a Tanh Activation Function with 2 layers and 32 inputs.

The 35 plasma proteomic biomarkers identified by LASSO were used to develop ML and DL models. We evaluated the performance of seven traditional ML methods [LR, NB, RF, GBM, KNN, XGBoost, and SVM algorithm (with three variations, namely, linear kernel, RBF kernel, and polynomial kernel)] and one DL method (namely, DNN) in the H2O-3 version 3.46.0.6 [48] with six variations, based on six different activation functions (Table 3). We selected this DL method as it has similar computational requirements with the traditional ML models compared. The criteria used to evaluate the performance of ML/DL models include accuracy, sensitivity, specificity, precision, F1-score, and AUC. Based on the accuracy and F1-score, the best model was the DL model with an activation function of “Rectifier With Dropout” with 2 layers and 32 proteomic biomarkers. This model achieved the highest accuracy of 0.995 and an F1 Score of 0.996. Figure 2 illustrates the variable importance of the DL model with “Rectifier With Dropout”. Based on accuracy, the second and the third best models were the DL model with an activation function of “Tanh With Dropout” with 3 layers and 32 and 33 proteomic biomarkers, respectively. Figure 3 illustrates the AUC curves in the validation data for models that used H2O for DL models using an activation function of “Rectifier With Dropout”, 2 layers and 32 inputs; an activation function of “Tanh With Dropout”, 3 layers, and 32 and 33 inputs, respectively; and an activation function of “Tanh” with 2 layers and 32 inputs.

Among the seven traditional ML models, the XGBoost revealed the best model with an accuracy of 0.986 and F1 Score of 0.985, and the second-best models were SVM and GBM with the same accuracy of 0.972 and the same F1 Score of 0.970 (Table 3).

### 2.4. Correlation Analysis

**Figure 4:**
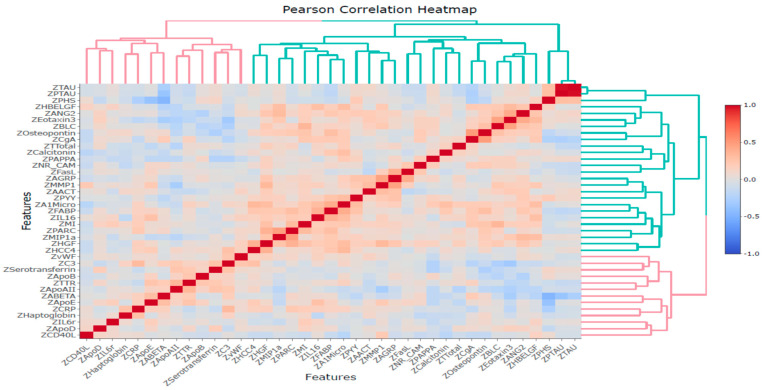
Pearson correlation heatmap for 39 variables based on Z-scores.

Pearson and Spearman correlation analyses were conducted among these 39 variables, including 35 proteomic biomarkers, PHS, and three CSF biomarkers (Aβ_42_, tTau, and pTau). The results revealed significant correlations between several proteins and PHS, tTau, pTau, or Aβ42 (Tables S2 and S3). Furthermore, based on Pearson’s correlation analysis, the proteins that significantly correlated with PHS were ApoD, ApoE, CgA, CRP, HBELGF, Osteopontin, and PYY proteins. The proteins significantly correlated with Aβ42 were ANG2, ApoAII, ApoE, C3, Calcitonin, CgA, CRP, Eotaxin3, HBELGF, MIP1a, MMP1, PAPPA, PYY, and TTR. The proteins significantly correlated with both pTau and tTau were CgA, FABP, Testosterone-Total, and TTR proteins. The Pearson correlation heatmap is illustrated in Figure 4.

### 2.5. Bioinformatics Analysis Using GO and KEGG Pathway Analyses

**Figure 5:**
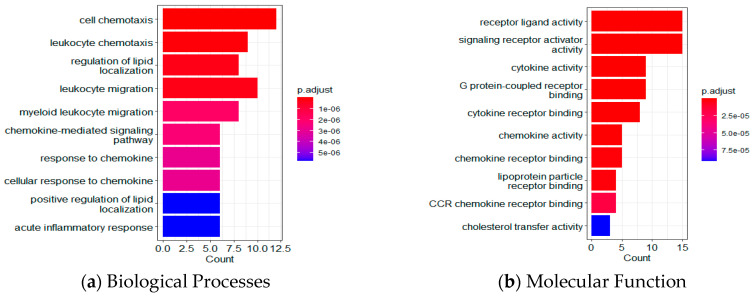

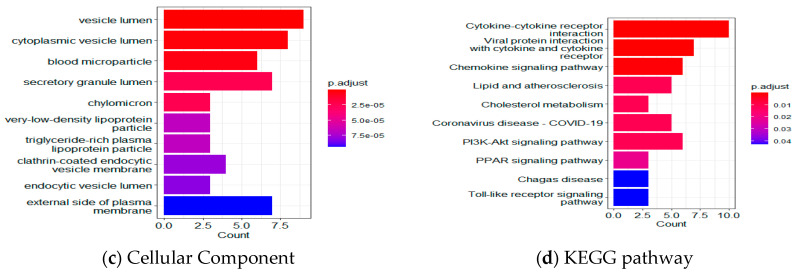
Functional enrichment analyses using GO and KEGG. Top 10 results for (**a**) biological processes; (**b**) molecular functions; (**c**) cellular components; and (**d**) the KEGG pathway.

Based on functional enrichment analyses of the 35 LASSO-selected proteomic markers using Gene Ontology (GO) and the Kyoto Encyclopedia of Genes and Genomes (KEGG), Figure 5 shows the top 10 proteins for biological processes (BP), molecular function (MF), cellular component (CC), and KEGG pathway. Table S4 lists the *p*-values, q-values, and number of involved genes for each pathway. In the BP category, target proteins were mainly involved in cell and leukocyte chemotaxis, regulation of lipid localization, and leukocyte migration. In the MF category, target proteins were primarily involved in receptor ligand activity, signaling receptor activator activity, cytokine/chemokine activity, G protein-coupled receptor binding, and cytokine/chemokine receptor binding. In the CC category, target proteins were mainly distributed in the vesicle lumen, cytoplasmic vesicle lumen, secretory granule lumen, the external side of the plasma membrane, and lipoprotein particles.

**Figure 6:**
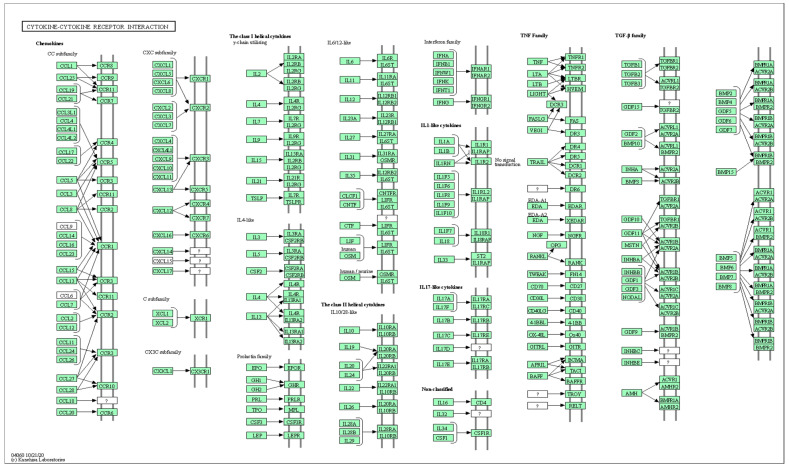
Explore the selected KEGG pathway of hsa04060 using the browse KEGG function. This figure shows 10 genes: CXCL13, CD40LG, CCL26, FASLG, CCR4, IL16, IL6R, CXCL9, CCL3, and CCL18.

KEGG pathway analysis revealed that several pathways were significantly enhanced, including cytokine-cytokine receptor interaction (KEGG pathway ID: hsa04060, q-value = 9.04 × 10^−7^, 10 genes, Figure 6 and Table S4), chemokine signaling pathway (q-value = 1.25 × 10^−3^, 6 genes), viral protein interaction with cytokine and cytokine receptor (q-value = 9.04 × 10^−7^, 7 genes), as well as lipid and atherosclerosis (q-value = 1.50 × 10^−2^, 5 genes), along with genes in cholesterol metabolism, PPAR, Toll-like receptor, and HIF-1 signaling pathways.

## 3. Discussion

The present study evaluated seven traditional ML models and six variations of a DL model in the prediction of MCI using plasma proteomic biomarkers. The LASSO algorithm identified 35 proteomic biomarkers, and the DL model with an activation function of “Rectifier With Dropout” with 2 layers and 32 inputs revealed the best model with the highest accuracy and F1 Score. Furthermore, the analysis of GO and KEGG pathways demonstrated that these identified proteins were involved in various crucial biological processes and pathways. These include cell chemotaxis, regulation of lipid localization, receptor ligand activity, signaling receptor activator activity, cytokine-cytokine receptor interaction, and viral protein interaction with cytokine and cytokine receptor.

The involvement of these proteins in these pathways suggests their potential contributions to the underlying mechanisms of MCI and provides valuable insights into the molecular processes implicated in the disease, as noted in a study that reported cerebrospinal fluid inflammatory analytes in relation to cognitive decline were best described by natural killer cell chemotaxis [51]. The studies using an animal model also demonstrated that cell chemotaxis was associated with female diabetic mice with cognitive impairment [52] and cognitive function in aged mice related to cytokine-cytokine receptor interaction [53]. These findings may have implications for understanding the pathogenesis of the MCI stage of AD and identifying potential therapeutic targets for intervention.

Feature selection is a critical step in ML not only to reduce the dimensionality of the feature space but also to reveal the most relevant features without losing too much information [45,46,47]. In prediction of AD, one study [38] used LASSO and selected 50 predictor proteins from a plasma sample to predict amyloid burden for preclinical AD. They obtained an AUC of 0.891, a sensitivity of 0.78, and a specificity of 0.77. Another study used LASSO to select a panel of non-redundant (non-correlated) protein biomarkers from brain tissues that could accurately diagnose AD [54]. One further study on CSF proteomics used the LASSO method to identify a panel of proteins that can differentiate AD-MCI from neurological controls while keeping the lowest possible dimensionality by eliminating the highly correlated proteins [41]. In a particular study [12], correlation-based feature selection and LASSO methods were used to develop biomarker panels from urine metabolomics samples. The panels were combined with an SVM model, resulting in 94% sensitivity, 78% specificity, and 78% AUC to distinguish healthy controls from AD. In another study [14], the LASSO algorithm was followed by two classification algorithms, namely SVM and RF, to identify eight top-ranked metabolic features that can differentiate stable MCI subjects, who are proceeding to AD, and AD patients, with an overall average accuracy of 73.5%. In the present study, the LASSO approach selected 35 proteomic biomarkers, most of which were significantly associated with MCI based on an independent *t*-test (Table S1).

Traditional ML technologies have been used to help in the diagnosis and prognosis of AD, such as LR, NB, RF, DT, GBM, and SVM [14,22,23,25,26,27,28,29,30]. In the past several years, DL techniques have become increasingly popular in AD research. One benefit of the DL approach over typical ML methods is that the reliability of DL techniques grows with the phases of learning. Some studies have shown that DL techniques were more accurate than traditional ML algorithms such as RF and SVM [25,27,34,35,36,37,55]. Furthermore, the H2O package in R supports the most widely used ML models and advanced models, such as multilayer feedforward ANNs [48]. Several studies have used the H2O automatic ML tools, including approaches such as LR, RF, GBM, DT, XGBoosting machine, and neural networks [56,57,58,59,60,61,62,63,64,65]. Several studies have used the H2O DL function for model building [56,59,64,66,67,68]. Recently, we developed H2O DL models using metabolomic biomarkers to predict AD [69]. Until now, no single study has used H2O DL to classify MCI using proteomic biomarkers. In this present study, we used grid search and evaluated seven traditional ML models and six variations of a DNN model. Findings suggest that the best model was the DL model with an activation function of “Rectifier With Dropout”. Our analysis showed that this variation of the DL model was also the overall best model (over all traditional ML methods and DL variations tested), reporting the highest accuracy and F1 Score in predicting MCI.

Protein biomarkers have the potential to inform disease progression (preclinical AD progressing to symptomatic AD), mechanisms, and endophenotypes. These biomarkers may not only individually show disease progression but also identify crucial biological pathways impacted by AD progression. Previous studies have provided evidence that CSF proteomic analyses have the ability to uncover changes in biological processes and pathways even at preclinical or MCI stages [15,16,17,18,20,21,22]. By examining the proteomic profiles of CSF, researchers have identified specific molecular alterations that are associated with the early stages of neurodegenerative diseases, including AD. These findings suggest that CSF proteomics hold great potential as diagnostic and prognostic tools for detecting and monitoring disease progression at its earliest stages, enabling interventions and treatments to be implemented in a more effective and timely manner. For example, one study found that 91% (325/335) of pathways in the KEGG database have been topics of scientific articles examining an association with AD, and 63% of pathway terms have a clear association with AD [70]. Another study reported that in subjective cognitive decline and the preclinical stage of AD, both glucose and amino acid metabolism pathways were dysregulated according to blood sample [71] and CSF metabolomics [72].

In the present study, the 35 plasma proteins selected through LASSO have revealed that several signaling pathways are altered in MCI, mostly in lipid metabolism, inflammation, and immune response (Figure 5 and Figure 6). For example, cytokine-cytokine receptor interaction and Toll-like receptor signaling pathways in the present study (Figure 5) have been shown in the top 10 KEGG pathways in AD and novel biomarkers reflecting neuroinflammation [41,73]. Furthermore, longitudinal studies incorporating serum analyses and brain neuropathology, along with the utilization of RF models, have provided valuable insights into the pathogenesis of AD [74,75,76] and highlighted the potential contributions of fatty acid, bile acid, cholesterol metabolism, and sphingolipid identified in the present study (Figure 5) to the development and progression of AD. Immune response and chemokine signaling pathway genes such as S100A12, CXCR4, and CXCL10 have been identified as potential markers for AD diagnosis and risk evaluation [77].

In addition, the Spearman and Pearson correlation analyses in the present study confirmed that the proteins in the above metabolic pathways in MCI are related to CSF biomarkers for AD (Tables S3 and S4). Therefore, our study supports the notion that multiple physiological pathways are involved in the onset and pathogenesis of MCI and AD. Among these pathways, a glycolysis enzyme (pyruvate kinase) and an immunological factor (MIF, macrophage inhibitory protein) are selected by several ML classifiers to be the most important feature next to the Tau proteins as CSF signatures for AD [21].

### Strengths, Limitations, and Future Directions

There are several strengths of this study. First, we performed feature selection using LASSO in the selection of plasma proteomic biomarkers. The LASSO approach selected 35 proteomic biomarkers, most of which were significantly associated with MCI based on an independent *t*-test (Table S1). Second, we used grid search to evaluate the accuracy of different models and compared seven traditional ML methods and 6 DL methods to predict MCI. Third, we performed correlation analyses to examine the complex relationships between these proteomic biomarkers, APOE genotypes, PHS, and clinical CSF biomarkers (Aβ42, tTau, and pTau). Fourth, we conducted pathway analyses using GO and KEGG to evaluate the functions of these proteins.

Additional strength for the present study is that plasma proteomic biomarkers were used in classifying the appearance of MCI. Most previous studies have focused on CSF proteomic analyses in AD [21,22,38,39,40,41,42]. For example, one method of CSF collection, brain imaging using CSF, is considered both high cost and high risk, and it remains unclear if these neuroimaging profiling biomarkers are specific to AD [12,13]. Another existing method of CSF collection is lumbar puncture (LP), an invasive procedure that carries the risk of adverse effects (i.e., infection) and requires a skilled clinician. Thus, a blood-based measure that accurately reflects the pathology of AD, ideally at the preclinical phase, has significant advantages in therapeutic trials and clinical decisions in prescribing Aβ-targeting drugs [38]. It has been shown that blood biomarkers could potentially aid early diagnosis and recruitment for trials [5,14,78,79]. One recent study suggests that urine-targeted proteomic biomarker has potential utility as a diagnostic screening tool in AD [80]. Several previous studies utilized plasma biomarkers [14,38,54,81], while other studies [74,75,82] focused on serum biomarkers in association with AD/dementia. Interestingly, both plasma and serum biomarkers yielded similar trends in their findings. A recent study [83] suggests that combining feature-selected serum and plasma biomarkers could be crucial for understanding the pathophysiology of dementia and developing preventive treatments. This highlights the urgent need for innovative analysis strategies, like ML, to effectively integrate serum and plasma biomarkers in MCI and AD research.

However, some limitations need to be acknowledged. First, the sample size is relatively small, especially the CN group (n = 57). The present study merged several components from ADNI-1, which originally had fewer CN individuals (400 individuals with MCI and 200 individuals with CN). After merging AOPE genotype, PHS, three clinical CSF biomarkers (Aβ42, tTau, and pTau), and plasma proteomic biomarkers, the sample size decreased because some measures were unavailable for certain individuals in ADNI-1. Although the ADNI data are longitudinal, the number of individuals of CN in the merged data are smaller in the follow-up; therefore, the present study relied on baseline data. Furthermore, the imbalanced data (57 CN and 182 MCI individuals) may affect the stability and generalization ability of the model, although the ROSE method was utilized to resample the data [50]. Moreover, education is a key component of cognitive reserve, and several studies have found that it has a relationship with MCI [10,84,85]. However, the present study did not show an association of education with MCI (Table 1). On the one hand, this could be due to phenotypic and genetic heterogeneity among the patients with cognitive impairment in different studies; on the other hand, the small sample size in the CN group may influence the results. In addition, the gender in the MCI group is not balanced. Although two-thirds of persons with Alzheimer’s are women [2], currently, it is unknown how gender plays a role in different pathways. In the future, independent large longitudinal data are required to assess the generalization ability of the model, consider using regularization techniques to reduce overfitting, and replicate the results.

Another limitation is that the present study only used proteomic biomarkers. Identification of biomarkers from proteomics is still in its very early stage due to many challenges in proteomics. For example, (1) the human genome encodes approximately 26,000–31,000 proteins, but current studies have limited coverage of the proteome; (2) unlike genomics, there is no standardized proteomic method analogous to polymerase chain reaction (PCR); and (3) proteomic methods remain relatively expensive [86,87,88,89,90]. Due to the complex mechanisms of the development of MCI and AD, future research should examine multiple “omics” data types and integrate multi-omics data by analyzing large-scale molecular data like genomics, transcriptomics, proteomics, and metabolomics data to understand the underlying biological mechanisms of MCI and AD at a comprehensive molecular level. The integration of data from multiple platforms will help to identify specific cellular pathways and new biomarkers altered with the progression of the disease, understand mechanisms, potential biomarkers, and therapeutic targets, and ultimately improve the diagnosis and treatment of AD [91,92,93,94,95]. Furthermore, integrating omics data needs advanced statistical methods such as correlation analyses, data mining, cluster analysis, mediation analysis, and more effective ML and DL tools.

In addition, it should be noted that the present study specifically aimed to evaluate traditional ML tools and a DNN model—a DL model included in the H2O package. Recently, DL models have been increasingly applied in the field of medical imaging. Several state-of-the-art (SOTA) models such as CNN, residual network (ResNet), Transformer, Mamba, diffusion models or combined models have been proposed [96,97,98,99,100,101,102]. For example, the CNNs and Vision Transformers (ViTs) are predominant paradigms; however, each has advantages and inherent limitations, whereas the emerging Mamba could combine the advantages of linear scalability and global sensitivity [98]. Another study proposed a novel network framework, MUNet, which could combine the advantages of UNet and Mamba [97]. A recent study proposed TabSeq, which applied tabular deep learning models to the ADNI data [103]. The present study focused on evaluation of DNN with traditional ML models because the DNN model is easy to use for one-dimensional proteomic data. The SOTA DL methods are computationally heavy and are beyond the scope of this paper. In the future, it will be interesting to evaluate DNN with other SOTA methods such as CNN-based methods, ResNet, Mamba, and Transformer-based models with a comprehensive comparison with modern DL models.

## 4. Materials and Methods

### 4.1. Dataset

This study used the Alzheimer’s Disease Neuroimaging Initiative (ADNI) database (adni.loni.usc.edu). The ADNI has the primary goal to test whether serial magnetic resonance imaging (MRI), positron emission tomography (PET), other biological markers, clinical, and neuropsychological assessments can be combined to measure the progression of mild MCI and early AD. In 2004, the ADNI began as a multicenter initiative to provide services to the United States and Canada. The ADNI is a longitudinal and multicenter study designed to develop clinical, imaging, genetic, and biochemical biomarkers for the early detection and tracking of AD. For this study, we merged several components of data from ADNI. There was an Institutional Review Board exemption for the current study due to secondary data analysis.

### 4.2. Measures

**Figure 7:**
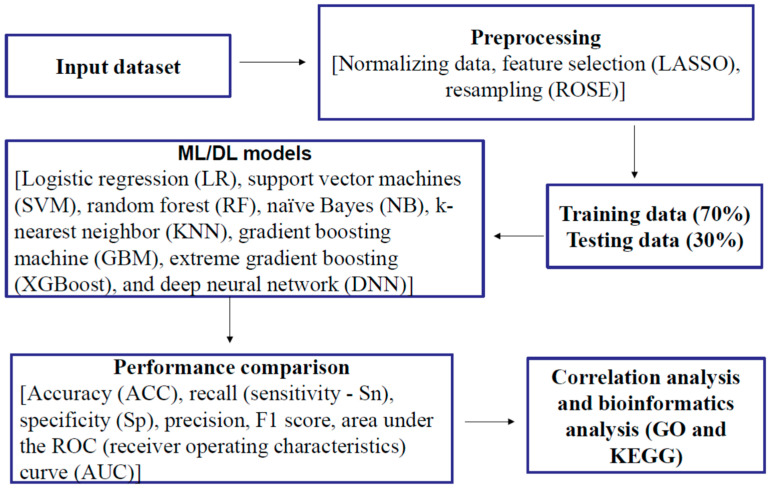
Research framework.

Demographic variables included age, gender, and educational levels. Gender has two levels (male or female). Age and education were continuous variables (years). *APOE-ε4* carriers were coded as individuals with at least one *ε4* allele (*ε4*/*ε4* designated as *APOE-ε4*-2, *ε4*/*ε3*, or *ε4*/*ε2* as *APOE-ε4*-1+), while non-carriers were defined as individuals with no *ε4* allele (*APOE-ε4*-0) (Table 1). Biological markers included polygenic hazard score (PHS) [104], which is based on AD-associated single nucleotide polymorphisms (SNPs) from previous genome-wide association studies (GWAS) data such as the International Genomics of Alzheimer’s Project and the Alzheimer’s Disease Genetics Consortium. PHS measures an individual’s risk of developing AD based on age and genetic markers. A total of 146 plasma proteomic biomarkers were from an ADNI subset of the dataset “Biomarkers Consortium Plasma Proteomics Project RBM multiplex data”. This study used the ADNIMERGE data, which consist of AD diagnosis, demographic variables, *APOE-ε4* genotype, and clinical CSF biomarkers (Aβ42, tTau, and pTau). After merging demographic variables, AD diagnosis, *APOE-ε4* allele-containing genotypes, PHS, clinical CSF biomarkers, and proteomic biomarkers, the total sample size was 335, including 96 with AD, 57 with cognitive normal (CN), and 182 with MCI. In the present study, we only analyzed data from MCI and CN individuals. Figure 7 shows the research framework, including data duration, the ML/DL process, correlation analysis, and bioinformatics analysis.

### 4.3. Feature Selection of Plasma Proteomic Biomarkers and Resampling

Before applying the feature selection methods of 146 plasma proteomic biomarkers, the protein levels were transformed to the Z score, as distributions of protein levels may be skewed. The Z-score was computed for each protein using the mean and standard deviation (SD). The Least Absolute Shrinkage and Selection Operator (LASSO) in the R package “glmnet” version 4.1-8 was used to perform feature selection using logistic regression [49]. LASSO is an embedded feature selection method used for ML and regression analysis. The LASSO method regularizes model parameter λ by shrinking the regression coefficients, reducing some of them to zero. Therefore, LASSO effectively performs feature selection, identifying and retaining only the most relevant features [49]. This makes LASSO useful for simplifying models, improving interpretability, and potentially preventing overfitting.

Considering the imbalanced data (57 with CN and 182 with MCI), the Random Over Sampling Example (ROSE) method was utilized, where the method = “both” was selected; both the minority class is oversampled with replacement, and the majority class is undersampled without replacement [50]. ROSE is a technique used to address class imbalance by generating synthetic data points. ROSE creates new samples by adding small perturbations to existing data points within the minority class [50]. This method uses smoothed bootstrap resampling to produce new instances. Another method—Synthetic Minority Over-sampling Technique (SMOTE)—works by selecting cases from the minority class and creating new samples along the line segments that connect these instances with their nearest neighbors [105]. Both ROSE and SMOTE were tried, and it was found that both improved model performance but did not rank as highly as oversampling or undersampling in the study [106]. The overall performance of ML was improved using either method, with ROSE performing better than SMOTE [107].

### 4.4. Traditional Machine Learning Methods

After feature selection, seven traditional ML algorithms were used, including LR, SVM, RF, NB, KNN, GBM, and XGBoost, to develop ML models to predict MCI. The Caret package version 7.0-1 in R [108] was used, incorporating RF version 4.7.1.1, NB version 1.0.0, GBM version 2.2.2, and XGBoost version 1.7.8.1. packages with tuning parameters. The data were split into 70% for training and 30% for validation. A grid search was employed on the training set to attain optimal values for the hyper-parameters. For each combination of hyper-parameters’ values, a 10-fold stratified cross-validation (CV) procedure was carried out to validate a model internally. The setting of parameters in each algorithm was briefly described as follows:

LR is the most widely used ML algorithm for binary outcomes in this study, MCI vs. CN. We implemented the method via “glmnet” in the Caret package. In the grid search, we set alpha = 0:1 and lambda = seq(0.001, 1, length = 20).

SVM is a method of computing hyperplanes that optimally separate data belonging to two classes. In this study, we used the nonlinear classification using the radial basis function (RBF) kernel [109]. In the grid search, we set sigma = c(0.01, 0.05, 0.1, 0.2, 0.5, 1) and C = c(0.01, 0.05, 0.1, 0.2, 0.5, 0.75, 1, 1.25, 1.5, 1.75, 2)).

The RF uses multiple decision trees and randomly selects a subset of variables and constructs many decision trees [110,111]. In the grid search, we set mtry = c(1:35) and ntree = 300, where the mtry parameter refers to the number of variables used in each random tree, while ntree refers to the number of trees that the forest contains.

The NB algorithm is a probabilistic ML model based on Bayes’ theorem with an assumption of independence between predictors, despite the fact that features in that class may be interdependent [112]. In the grid search, we set laplace = c(0, 0.5, 1, 2, 3, 4, 5) and adjust = c(0.75, 1, 1.25, 1.5, 2, 3, 4, 5).

KNN is a simple ML algorithm that calculates the average of the numerical target of the K nearest neighbors. KNN is based on a clustering algorithm with supervised learning [113]. KNN is more suitable for low-dimensional data with a small number of input variables. In the grid search, we set k = 1:35.

The GBM is a boosting algorithm and an ensemble model where many weak classification tree models are converted into one single strong model to produce prediction [49]. In the grid search, we set interaction.depth = c(1, 2, 3, 4, 5, 6), n.trees = (1:30)*50, shrinkage = c(0.005, 0.025, 0.05, 0.075, 0.1, 0.25, 0.5), and n.minobsinnode = c(5, 10, 15, 20).

XGBoost is a boosting tree-based ML framework and uses the CART (Classification and Regression Tree) and trains the trees serially and interactionally rather than in parallel and independently [114]. In the grid search, we set the nrounds = c(100, 200, 300), max_depth = c(6, 10, 20), colsample_bytree = c(0.5, 0.75, 1.0), eta = c(0.1, 0.3, 0.5), gamma = c(0, 1, 2), min_child_weight = c(1, 3, 5), and subsample = c(0.5, 0.75, 1.0).

### 4.5. Deep Learning Methods

**Figure 8:**
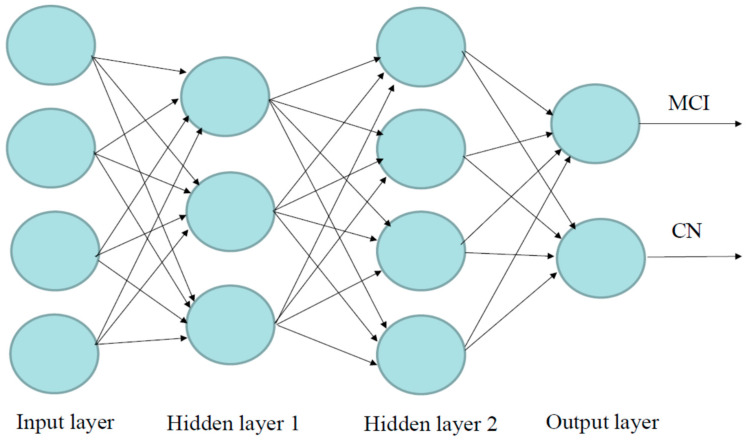
Visualization of the artificial neural network model with four inputs, two hidden layers, and output. Neurons are illustrated by circles. The neurons in the input layer receive values and propagate them to the neurons in the middle layer (“hidden layer”) of the network.

The DL in the “H2O” package is used to develop the DL models, which are on a multi-layer feedforward artificial neural network (ANN), also known as a deep neural network (DNN) or multi-layer perceptron (MLP). Figure 8 illustrates an ANN with two hidden layers, four input variables/neurons, and output. The data were split into 70% for training and 30% for validation. We used grid search and compared six different activation functions for the DNN: “Rectifier”, “RectifierWithDropout”, “Maxout”, “MaxoutWithDropout”, “Tanh”, and “TanhWithDropout”, with settings of the hidden layer as 1 to 3, n-fold cross-validation with n = 10, epochs = 100, input_dropout_ratio = 0.2, and l1 = 1 × 10^−6^. Search criteria included strategy = “RandomDiscrete”, max_models = 100, max_runtime_secs = 900, stopping_tolerance = 0.001, and stopping_rounds = 15. The models that return higher accuracy were chosen. Then, we focused on several DL models with higher accuracy and compared models with 1–3 hidden layers and different numbers of neurons.

### 4.6. Performance of Machine Learning and Deep Learning Models

The confusion matrix is illustrated in Table 4. To evaluate different models, the following performance measures were used: accuracy (ACC), recall (also called sensitivity—Sn), specificity (Sp), precision (positive predictive value—PPV), F1-score, and AUC (area under the receiver operating characteristic (ROC) curve). These measures are defined as follows:

(1) $$Accuracy=\frac{TP+TN}{TP+TN+FP+FN}$$

(2) $$Recall=\frac{TP}{TP+FN}$$

(3) $$Specificity=\frac{TN}{TN+FP}$$

(4) $$Precision=\frac{TP}{TP+FP}$$

(5) $$F1\;Score=\frac{2\;x\;Precision\;x\;Recall}{Precision+Recall}$$

Accuracy (ACC) is the ratio of correctly classified observations to the total number of observations. Recall (sensitivity—Sn) is the ratio of correctly predicted positive observations to all observations in the actual class—MCI. Specificity (Sp)—the ratio of correctly predicted negative observations to all observations in the actual class—CN. Precision (positive predictive value—PPV) is the ratio of correctly predicted positive observations to the total predicted positive observations. F1-Score is a harmonic mean that combines both recall and precision. The AUC is the measure of the ability of a classifier to distinguish between classes and is used as a summary of the receiver operating characteristic (ROC) curve.

### 4.7. Statistical Analysis

Descriptive statistics such as counts and proportions were used for categorical variables. Continuous variables were presented in the form of mean ± standard deviation (SD). A Chi-square test was used to detect the associations of categorical variables with MCI vs. CN. An independent *t*-test was used to examine the differences in continuous variables between MCI and CN. Pearson and Spearman correlation analyses were conducted to examine the relationships between the plasma proteomic biomarkers, PHS, and three CSF biomarkers (Aβ_42_, tTau, and pTau). Statistical analyses were performed using SAS 9.4 (SAS Institute, Cary, NC, USA).

### 4.8. Bioinformatic Analysis

The R package “ClusterProfiler” version 4.1.5.1 was used to perform Gene Ontology (GO) enrichment (http://www.geneontology.org, accessed on 30 July 2024) [115] and Kyoto Encyclopedia of Genes and Genomes (KEGG) pathway (http://www.genome.jp/kegg/pathway.html, accessed on 30 July 2024) [116] analyses of proteins to evaluate whether the targeted proteins were involved in important biological processes. Results were visualized by the R package “ggplot2” version 3.5.1 [117] in R 4.4.3 (R Core Team, Vienna, Austria).

### 4.9. Power Analysis

An independent samples *t*-test was used to compare the means of continuous variables between the two groups (MCI vs. CN). Using G.Power [118,119], assuming α = 0.05, Cohen’s d = 0.50 (moderate effect), and a sample size for MCI and CN being 182 and 57, respectively, the power can reach 92.96%.

## 5. Conclusions

In the present study, we evaluated seven traditional ML tools with six variations of the DNN model in the classification of MCI. We found the DL model with an activation function of “Rectifier With Dropout” with 2 layers and 32 proteomic biomarkers revealed the best model with the highest accuracy and F1 Score. The 35 proteins selected by LASSO are involved in important biological processes and pathways. Some of the proteins are correlated with the genetic biomarker *APOE-ε4*, PHS, and clinical CSF biomarkers (Aβ42, tTau, and pTau). These biomarkers and pathway analyses may help with early diagnosis, prognostic risk stratification, and early treatment interventions for individuals at risk for MCI and AD. The DL algorithm showed a higher performance compared to traditional ML models, and the DL model developed in this study might play a critical role in preventing the risk of MCI.

## Figures and Tables

**Table 1:** Descriptive statistics.

	CN	MCI	
Variable	(Mean ± SD or n)	(Mean ± SD or n)	t/χ^2^, *p*
Age	75.2 ± 5.8	74.3 ± 7.6	0.98, 0.3269
Gender (n)			
Male	29	121	4.52, 0.0334
Female	28	61	
Education	15.6 ± 2.7	15.8 ± 3.0	−0.55, 0.5796
*APOE*-*ε4* allele			
0	52	84	35.96, <0.0001
1+	5	98	
PHS	−0.49 ± 0.9	0.51 ± 1.12	−6.79, <0.0001
Aβ42	1452.8 ± 263.4	821.2 ± 421.1	13.49, <0.0001
tTAU	225.7 ± 73.1	313.0 ± 141.1	−6.12, <0.0001
pTAU	19.9 ± 6.5	30.9 ± 15.7	−7.70, <0.0001

Abbreviations: CN: cognitive normal; MCI: mild cognitive impairment; PHS: polygenic hazard score; SD: standard deviation. The *p*-value is based on the Chi-square test or independent samples *t*-test.

**Table 2:** Feature selection using Least Absolute Shrinkage and Selection Operator (LASSO).

Package	Feature Selection Algorithm	Extracted Variables
glmnet in R	Least Absolute Shrinkage and Selection Operator (LASSO)	35 variables: A1Micro, AACT, AGRP, ANG2, ApoAII, ApoB, ApoD, ApoE, BLC, C3, Calcitonin, CD40L, CgA, CRP, Eotaxin3, FABP, FasL, Haptoglobin, HBELGF, HCC4, HGF, IL16, IL6r, MI, MIP1a, MMP1, NR_CAM, Osteopontin, PAPPA, PARC, PYY, TTotal, Serotransferrin, TTR, vWF

**Table 3:** Machine learning and comparison of performance.

Model	Variation	Accuracy	Sensitivity (Recall)	Specificity	Precision	F1-Score	AUC
SVM	RBF Kernel	0.972	0.941	1.000	1.000	0.970	0.999
LR		0.958	0.941	0.973	0.970	0.955	0.998
RF		0.958	0.912	1.000	1.000	0.954	0.998
GBM		0.972	0.941	1.000	1.000	0.970	0.999
XGBoost		0.986	0.971	1.000	1.000	0.985	0.997
KNN		0.845	0.765	0.919	0.897	0.826	0.962
NB		0.944	0.882	1.000	1.000	0.937	0.982
DNN	Rectifier-c(35)	0.984	1.000	0.957	0.975	0.986	0.990
	Rectifier-c(32,32,32)	0.989	0.992	0.989	0.990	0.991	0.992
	Maxout-c(35)	0.960	0.957	0.973	0.963	0.956	0.973
	Tanh-c(35)	0.990	0.992	0.973	0.986	0.988	0.995
	Tanh-c(32,32)	0.990	1.000	0.979	0.980	0.990	0.995
	Rectifier With Dropout-c(35)	0.977	1.000	0.953	0.963	0.979	0.989
	Rectifier With Dropout-c(30,30)	0.989	0.978	1.000	1.000	0.988	0.992
	Rectifier With Dropout-c(32,32)	0.995	1.000	0.983	0.993	0.996	0.997
	Maxout With Dropout-c(35)	0.983	0.992	0.980	0.978	0.984	0.990
	Tanh With Dropout-c(35)	0.989	1.000	0.973	0.980	0.990	0.997
	Tanh With Dropout-c(34,34)	0.988	1.000	0.980	0.978	0.988	0.996
	Tanh With Dropout-c(32,32,32)	0.994	1.000	0.990	0.988	0.993	0.999
	Tanh With Dropout-c(33,33,33)	0.994	1.000	0.990	0.988	0.993	0.996

Abbreviations: SVM: support vector machine; RBF: radial basis function; LR: logistic regression; RF: random forest; GBM: gradient boosting machines; XGBoost: Extreme Gradient Augmentation; KNN: k-nearest neighbor; NB: naïve Bayes; DNN: deep neural network; AUC: area under the ROC (receiver operating characteristics) curve.

**Table 4:** Confusion matrix.

Confusion Matrix		Predicted Class	
		MCI	CN
Actual class	MCI	TP	FN
	CN	FP	TN

TP is the number of true positives—the model correctly predicted an MCI outcome (the actual outcome was MCI), TN is the number of true negatives—the model correctly predicted a CN outcome (the actual outcome was CN), FP is the number of false positives—the model incorrectly predicted an MCI outcome (the actual outcome was CN), and FN is the number of false negatives—the model incorrectly predicted a CN outcome (the actual outcome was MCI).

## Data Availability

Data used in the preparation of this article were obtained from the Alzheimer’s Disease Neuroimaging Initiative (ADNI) database (adni.loni.usc.edu).

## Supplementary Materials

The following supporting information can be downloaded at: https://www.mdpi.com/article/10.3390/ijms26062428/s1.

## Abbreviations

The following abbreviations are used in this manuscript:

ADNI	Alzheimer’s Disease Neuroimaging Initiative
AD	Alzheimer’s Disease
MCI	Mild Cognitive Impairment
CN	Cognitive normal
DL	Deep learning
ML	Machine learning
ROSE	Random Over Sampling Example
CSF	Clinical cerebrospinal fluid
GO	Gene Ontology
KEGG	Kyoto Encyclopedia of Genes and Genomes
LR	Logistic regression
DT	Decision trees
GBM	Gradient boosting machines
GLM	General linear model
SVM	Support vector machine
RF	Random forest
LASSO	Least Absolute Shrinkage and Selection Operator
RBF	Radial basis function
NB	Naïve Bayes
ANN	Artificial neural network
MLP	Multi-layer perceptron
DNN	Deep Neural Network
SAE	Stacked autoencoder
CNN	Convolutional neural network
SOTA	State-of-the-art (SOTA)
ResNet	Residual network
APOE	Apolipoprotein E
AUC	Area under the ROC (receiver operating characteristics) curve
PHS	Polygenic hazard score
SNP	Single nucleotide polymorphism
GWAS	Genome-wide association studies
SD	Standard deviation
Aβ	Amyloid-β
tTau	pathologic tau
pTau	Phosphorylated tau
MRI	Magnetic resonance imaging
PET	Positron emission tomography
ACC	Accuracy
Sn	Sensitivity
Sp	Specificity
PPV	Positive predictive value
TP	Number of true positives
TN	Number of true negatives
FP	Number of false positives
FN	Number of false negatives
BP	Biological processes
MF	Molecular function
CC	Cellular component
